# Hesitancy about COVID-19 vaccination among pregnant women: a cross-sectional study based on the health belief model

**DOI:** 10.1186/s12884-022-04941-3

**Published:** 2022-08-02

**Authors:** Mojgan Firouzbakht, Hamid Sharif Nia, Fatemeh Kazeminavaei, Pegah Rashidian

**Affiliations:** 1 Department of Nursing- Midwifery, Comprehensive Health Research Center, Babol Branch, Islamic Azad University, Babol, Iran; 2 Amol Faculty of Nursing and Midwifery, Traditional and Complementary Medicine Research Center, Addiction Institute, Mazandaran University of Medical Science, Sari, Iran; 3 Student Research committee, Guilan University of Medical Sciences, Rasht, Iran

**Keywords:** COVID-19, Pregnant women, Hesitancy about COVID-19 vaccination, Iran

## Abstract

**Background:**

Pregnant women are at high risk for affliction by coronavirus disease 2019 (COVID-19). Vaccination is a main strategy to prevent and manage the COVID-19 pandemic. However, hesitancy about COVID-19 vaccination (HACV) is a major public health threat and a major barrier to herd immunity. The aim of the study was to evaluate pregnant women’s HACV based on the Health Belief Model (HBM).

**Methods:**

This cross-sectional study was conducted in 2021–2022. Participants were 352 pregnant women selected from several healthcare centers in the north of Iran. Instruments for data collection were a demographic questionnaire, a COVID-19 Knowledge Questionnaire, a COVID-19 Health Belief Questionnaire, and a question about HACV. Logistic regression analysis was used to assess the effects of the study variables on HACV.

**Results:**

The rate of HACV was 42.61%. In the regression model, the three factors of perceived benefits (aOR: 0.700; 95% CI: 0.594 to 0.825), cues to action (aOR: 0.621; 95% CI: 0.516 to 0.574), and history of reproductive problems (aOR: 2.327; 95% CI: 0.1.262 to 4.292) had significant effects on HACV (*P* <  0.001).

**Conclusion:**

HACV is highly prevalent among pregnant women. The perceived benefits and cues to action components of HBM have significant effects on pregnant women’s HACV, while the perceived threat component has no significant effect on it. HBM is a good model to explain HACV among pregnant women. Educational interventions are necessary to improve pregnant women’s awareness of the risks of COVID-19 for them and their fetus.

## Background

The coronavirus disease 2019 (COVID-19) outbreak started in December 2019 and rapidly turned into a pandemic affecting many people throughout the world. Up to February 2022, the number of patients with COVID-19 and the number of COVID-19-related deaths were more than 414 million and six million in the world [1] and more than six million and 134,000 in Iran, respectively (behdasht.gov.ir). In May 2020, the World Health Organization recognized mass vaccination as a main strategy to prevent and manage the COVID-19 pandemic [2]. Subsequently, more than 125 types of COVID-19 vaccine were produced and eighteen of them were approved in different trials [3]. Vaccination demonstrated protection against COVID-19 infection, hospitalization and death [4]. As the results of studies showed that decline in vaccine effectiveness over time [5, 6], need to booster shots in vulnerable population groups be recommended [7].

Pregnant women are at high risk for affliction by COVID-19 [8]. Compared with their non-pregnant counterparts, pregnant women with COVID-19 are more likely to need hospitalization in intensive care unit and mechanical ventilation and are more likely to experience COVID-19-related death [9] and pregnancy complications such as preterm birth [10]. The COVID-19 pandemic was also associated with varying levels of stress and anxiety [11] which increase the risk of pregnancy complications such as preeclampsia, depression, nausea and vomiting, preterm birth, low birth weight, and low Apgar score [12]. Therefore, pregnant women should receive timely COVID-19 vaccine [13, 14]. Nonetheless, none of the existing trials into COVID-19 vaccine included pregnant women and there are limited data and some levels of uncertainty about the effects of COVID-19 on pregnancy [15].

Uncertainty about the effects of COVID-19 on pregnancy can negatively affect pregnant women’s decision to receive COVID-19 vaccine and lead to hesitancy about vaccination [16]. According to the World Health Organization, hesitancy about vaccination is delay in or refusal of receiving vaccine despite its availability and accessibility [17]. A study in sixteen countries reported that the rate of COVID-19 vaccine acceptance was 52% among pregnant women and 73.4% among non-pregnant women [16]. Another study on pregnant and breastfeeding women in six European countries reported that the rate of hesitancy about COVID-19 vaccination (HACV) was 40–50% [18].

Hesitancy about vaccination is a complex phenomenon affected by many different personal or contextual factors [19] such as social context, vaccine availability, satisfaction with vaccine [17], perceived vaccine safety [17, 20], perceived benefits of vaccination [21], religious beliefs [22, 23], lack of knowledge [24], and attitude towards vaccine [25]. HACV is also affected by socio-demographic characteristics such as age, gender, educational level, insurance, trust in governmental information, perceived susceptibility to COVID-19, perceived benefits and complications of vaccination, and attitude towards vaccine [26].. HACV is considered as a public health threat [27].

A study in ten countries in Asia, Africa, and South America showed that poor beliefs of vaccination benefits and beliefs that new vaccines are riskier were related with females, Muslim, having a non-healthcare-related job and not receiving a flu vaccination during past 12 months [28]. Also, political affiliation, and the perceived threat of getting infected with COVID-19 in the next 1 year was predicted vaccine hesitancy [29].

Factors contributing to health-related behaviors, such as vaccination, can better be assessed using behavior-related models such as the Health Belief Model (HBM). HBM is one the most commonly used models for understanding health-related behaviors and has been used to assess vaccination-related behaviors [30–33]. Compared with other models on health-related behaviors, HBM was specifically developed for preventive health-related researches [34]. This model had four main components, namely perceived susceptibility (i.e., perceived likelihood of affliction by a disease), perceived severity (i.e., the severity of disease consequences), perceived benefits (i.e., the benefits of preventive measures or treatments), and perceived barriers (i.e., the barriers to the use of preventive measures) [35]. Later, three other components, namely demographic characteristics, self-efficacy, and cues to action, were added to the model [36]. HBM holds that individuals are more likely to engage in disease preventive behaviors such as vaccination if they perceive that they are susceptible to the disease, the disease is severe, behaviors are beneficial, and there are minimal barriers to the behaviors [30, 37, 38]. Cues to action respecting vaccination also refer to healthcare providers’ recommendations or health education messages [39].

Despite the importance of vaccination to COVID-19 prevention and management as well as the low rate of vaccination among pregnant women, there are limited data about pregnant women’s acceptance of COVID-19 vaccine. Consequently, the present study was conducted to narrow this gap. The question of this study was:

Is the HBM construct associate with vaccine hesitancy in Iranian pregnant women?

## Methods

### Design

**Fig. 1:**
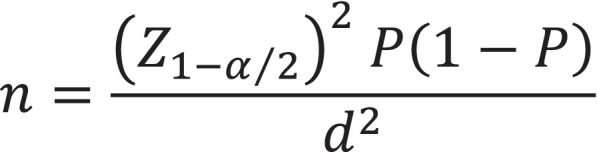
Sample size formula

This cross-sectional study was conducted from October 23, 2021 to January 4, 2022, in Mazandran province, north of Iran. Sample size was calculated with a hypothetical vaccine acceptance rate of 54%, based on results of a systematic review and meta- analysis on acceptance vaccines COVID_19 in pregnant women that was conducted in Iran in 2022 [40], a confidence level of 0.95, a *d* of 0.1*P*, and a probable attrition rate of 15%. Sample size calculation formula (Fig. 1) revealed that 350 participants were needed.

Study setting was leading public healthcare centers in the north of Iran. These centers were Imam Ali (PBUH) hospital, Amol, Al-Zahra hospital, Rasht, and several leading public healthcare centers in Amoal and Babol, Iran. For the selection of public healthcare centers, Amol and Babol cities were divided into four hypothetical geographical areas (i.e., north, east, west, and south) and then, one center with the highest number of referring pregnant women was selected from each area. The pregnant women who were included in the study had a complete health file in selected health care centers. The pregnant women were recruited to the study through convenience sampling. Sampling was done by midwives of the health care centers. Midwives in the study setting provided the study instruments to participants and asked them to complete them through the self-report method. Eligibility criteria were pregnancy, age over eighteen years, basic literacy skills, Iranian nationality, and agreement for participation.

### Instruments

Instruments for data collection were a demographic questionnaire, a COVID-19 Knowledge Questionnaire, a COVID-19 Health Belief Questionnaire, and a question about HACV. The demographic questionnaire had items on age (year), educational level (diploma or less, associate/bachelor’s, master’s or higher), occupation (housewife, employed), number of pregnancies, gestational age (week), pregnancy complications in the current pregnancy (yes, no), history of chronic diseases (yes, no), and history of flu vaccination (yes, no).

The COVID-19 Knowledge Questionnaire was a researcher-made questionnaire developed based on the protocols of the World Health Organization and the Ministry of Health and Medical Education of Iran for COVID-19 prevention and management. This questionnaire had ten items with three possible answers, namely “Yes” (scored 1), “No” (scored 0), and “I don’t know” (scored 0). Therefore, the total possible score of the questionnaire was 0–10, with higher scores showing better COVID-19 knowledge. The total score was interpreted based on the tertiary rule of thumb [27] as follows: scores less than 4: limited knowledge; scores 4–7: moderate knowledge; and scores more than 7: great knowledge. We determined the validity and reliability of the questionnaire. The validity of the questionnaire was confirmed through the face and content validity assessment methods. Eleven experts rated the items, and the content validity indices (CVI) of the questionnaire were calculated to be 0.81. The Cronbach’s alpha of the questionnaire was 0.82, indicating internal consistency.

The COVID-19 Health Belief Questionnaire was also a researcher-made questionnaire developed based on the studies into infectious diseases which can be prevented through vaccination [27, 41]. It has six main dimensions, namely perceived susceptibility (two items), perceived severity (two items), perceived barriers (three items), perceived benefits (three items), cues to action (two items), and self-efficacy (three items). Perceived susceptibility and perceived severity were considered as perceived threat. Items were scored on a five-point scale from 1 (“Completely disagree) to 5 (“Completely agree”). Items in the perceived barriers dimension were reversely scored. The sum of the items of each dimension was considered as the total score of that dimension. Twelve experts in reproductive health, nursing, and education approved the face and content validity. However, it has no valid and reliable Persian version. Construct validity was evaluated through with exploratory factor analysis. Exploratory factor analysis (EFA) is a multivariate statistical technique that describer the relationship of some observed variables by a relatively number of factors. In maximum likelihood exploratory factor analysis, KMO test value was 0.854 and Bartlett’s test value was 1673.202 (*P* <  0.001), indicating the sampling was adequate. Total variances extracted were 55.71%. The Cronbach’s alpha values of the questionnaire and its dimensions were respectively 0.81 and 0.76 to 0.88, which confirmed its acceptable reliability.

HACV was assessed through the following closed-ended question, “How much are you willing to receive COVID-19 vaccine?” The five possible answers to this question were,

1. “I accept receiving any vaccine recommended by the healthcare center”;
2. “I accept receiving COVID-19 vaccine but have hesitancy about it”;
3. “I accept receiving only a certain type of COVID-19 vaccine”;
4. “I will accept receiving COVID-19 vaccine after pregnancy”;
5. “I do not accept receiving COVID-19 vaccine” [42].

Selecting items 3–5 was considered as HACV [27].

### Data analysis

The SPSS program (v. 22.0) was employed to analyze the study data. The Chi-square and the independent-sample *t* tests were used to determine the relationship of HACV with other study variables and the logistic regression analysis was used to determine their odds. We applied the multiple logistic regression models to estimate the regression coefficients of vaccine hesitancy in association with subscales of HBM construct. In this analysis, we considered the total score of sub scales of HBM construct as independent variables and vaccine hesitancy as a dichotomous variable with the two levels of yes / no as dependent variables. The additional multiple regression models for adjustment of regression coefficients were carried out. The effect of potential confounding such as knowledge, history of medical diseases, and history of reproductive problems was adjusted, and the 95% confidence interval (CI) of coefficients was estimated. The *p*-value less than 0.05 were considered as a significant level.

## Results

An overall of 380 sets of instruments were distributed among 380 eligible participants and 352 completely filled instruments were included in final analysis (response rate = 0.92%). On average, participants’ age and COVID-19 knowledge were 30.13 ± 5.52 years and 5.849 ± 1.685, respectively. Most participants aged 20–35 years (71.3%), were in the third trimester of their pregnancy (70.7%), and had moderate COVID-19 knowledge (69.7%), while 30% of them had pregnancy complications (such as hypertension, bleeding, and preterm birth) and 16.5% of them had received the influenza vaccine (Table 1).

**Table 1:** Participants’ demographic characteristics and their relationships with HACV

Characteristics		N (%)	HACV		χ2	*P* Value
			No, N (%)	Yes, N (%)		
Age (Years)	< 20	17 (4.8)	7 (3.5)	10 (6.7)	2.184	0.336
	20–35	277 (78.7)	163 (58.8)	114 (76)		
	> 35	58 (16.5)	32 (15.8)	26 (17.3)		
Educational level	Diploma or less	163 (46.6)	94 (46.5)	69 (46)	1.464	0.481
	Associate/Bachelor’s	150 (42.8)	83 (41.1)	68 (45.3)		
	Master’s or higher	37 (10.6)	25 (12.4)	13 (8.7)		
Occupation	Housewife	284 (80.7)	166 (82.2)	118 (78.7)	0.681	0.409
	Employed	68 (19.3)	36 (17.8)	32 (21.3)		
Place of residence	Urban areas	256 (72.7)	145 (71.8)	111 (74)	0.213	0.644
	Rural areas	96 (27.3)	57 (28.2)	39 (26)		
Gestational age	First trimester	37 (10.3)	22 (10.9)	15 (10)	0.919	0.632
	Second trimester	66 (18.8)	41 (20.3)	25 (16.7)		
	Third trimester	249 (70.9)	139 (68.8)	110 (74.3)		
Disease history of	Yes	118 (33.4)	61 (30.2)	57 (38)	2.351	0.125
	No	234 (66.6)	141 (82.2)	93 (62)		
History of reproductive problems	Yes	109 (30.8)	53 (26.2)	56 (37.3)	4.957	0.026*
	No	243 (69.2)	149 (73.8)	94 (62.7)		
Influenza vaccination	Yes	58 (16.5)	39 (19.3)	19 (12.7)	2.758	0.096
	No	294 (83.5)	163 (80.7)	131 (87.3)		
COVID-19 knowledge	Limited	39 (11.1)	20 (9.9)	19 (12.7)	9.074	0.011*
	Moderate	245 (69.6)	132 (65.3)	113 (75.3)		
	Great	68 (19.3)	50 (24.8)	18 (12)		

*: *p* <  0.05

More than two fifth of participants accepted receiving any vaccine (42.9%), while 10.2% reported that they would not accept receiving any vaccine. The rate of HACV was 42.613%. In other words, 150 participants had HACV and the remaining 202 participants (57.386%) did not have HACV (Table 2). HACV had significant relationship with pregnancy complications (*P* = 0.026) and COVID-19 knowledge (*P* = 0.011) (Table 1).

**Table 2:** The prevalence of HACV

Decision about COVID-19 vaccination	N	%
I do not accept receiving COVID-19 vaccine	36	10.2
I accept receiving any vaccine recommended by the healthcare center	151	42.9
I accept receiving COVID-19 vaccine but have hesitancy about it	51	14.8
I accept receiving only a certain type of COVID-19 vaccine	8	2
I would accept receiving COVID-19 vaccine after pregnancy	106	30.1

There were significant differences between participants with HACV and participants without HACV respecting the mean scores of all components of HBM, namely perceived threat, perceived barriers, perceived benefits, cues to action, and self-efficacy (*P* <  0.05) (Table 3).

**Table 3:** The mean ± SD of subscale of HBM according to the HACV

HBM component	HACV		*t*	Mean difference	*P* value
	No	Yes			
Perceived threat	15.816 ± 2.931	15.113 ± 3.180	2.082	0.703	0.038*
Perceived barriers	10.682 ± 2.445	9.673 ± 2.307	3.810	1.009	< 0.001**
Perceived benefits	11.284 ± 2.198	8.924 ± 2.23	9.550	2.360	< 0.001**
Cues to action	7.914 ± 1.553	5.718 ± 2.091	10.909	2.195	< 0.001**
Self-efficacy	12.523 ± 2.033	10.527 ± 2.624	7.805	1.985	< 0.001**

*P < o.o5, **P <  0.001

Logistic regression analysis was conducted for evaluation of odds of HACV. According to the simple regression analyses including the HBM factors, COVID-19 knowledge and History of reproductive problems (Table 4), the participants were more likely to be vaccine hesitant if they had high perceived barriers to vaccination (OR 1.340, 95% CI 0.863 to 2.367, *p* = 0.032). The participants were less likely to be vaccine hesitant if they had high perceived benefits of vaccination (OR 0.609, 95% CI 0.534 to 0.694, *p* <  0.001), had high perceived threat (OR 0.927, 95% CI 0.862 to 0.996, *p* = 0.039), had high self-efficacy for vaccination (OR 0.684, 95% CI 0.611 to 0.767, *p* <  0.001), and high cues to action for vaccination (OR 0.520, 95% CI 0.444 to 0.609, *p* <  0.001), and high COVID-19 knowledge (OR 0.585, 95% CI 0.392 to 0.875, *p* = 0.009).

**Table 4:** Adjusted and unadjusted regression coefficients of HACV in relation to subscales of HBM and COVID-19 Knowledge, Disease history, and history of reproductive problems

HBM Components	HACV							
	Model 1^a^				Model 2^b^			
	B	COR	P	CI	B	aOR	P	CI
Perceived threat	−0.076	0.927	0.039	0.862 to 0.996				
Perceived barriers	0.179	1.340	0.032	0.863 to 2.367				
Perceived benefits	−0.496	0.609	< 0.001	0.534 to 0.694	− 0.357	0.700	< 0.001	0.594 to 0.825
Cues to action	−0.654	0.520	< 0.001	0.444 to 0.609	−0.477	0.621	< 0.001	0.516 to 0.747
Self-efficacy	−0.379	0.684	< 0.001	0.611 to 0.767				
COVID-19 knowledge	−0.535	0.585	0.009	0.392 to 0. 875				
Disease history	0.348	1.417	0.126	0.907 to 2.213				
History of reproductive problems	0.516	1.675	0.029	1.062 to 2.642	0.845	2.327	0.007	1.262 to 4.292

^a^Model1: Unadjusted model

^b^Model 2: Adjusted model

The overall model was statistically significant when compared to the null model, (χ^2^ (3) = 117.525, *p* <  0.001), explained 43.5% of the variation of survival (Nagelkerke R^2^) and correctly predicted 75.3% of cases. However, the multiple logistic regression analysis revealed that HACV had significant relationship only with perceived benefits (aOR: 0.700; 95% CI: 0.594 to 0.825; *P* <  0.001), cues to action (aOR: 0.621; 95% CI: 0.516 to 0.574; *P* <  0.001), and history of reproductive problems (aOR: 2.327; 95% CI: 0.1.262 to 4.292; *P* <  0.001) (Table 4).

## Discussion

The aim of this study was to evaluate pregnant women’s HACV based on HBM. Study findings also indicated that perceived benefits and cues to action had significant relationship with HACV. Several previous studies also reported the same finding [26, 27, 43]. This study coincided with the sixth wave of COVID-19 in Iran and vaccination acceptance rate was much higher than the rate at the beginning of vaccination [44]. A study reported that individuals’ awareness of the reduced risk of COVID-19 after vaccination significantly improved vaccination rate [45]. Although the rate of COVID-19 vaccination has not significantly increased among pregnant women compared with the general public, the Centers for Disease Control and Prevention reported that concern over affliction by COVID-19 has increased among non-vaccinated individuals [44]. Strong evidence exists respecting the usefulness of COVID-19 vaccination in preventing affliction by severe COVID-19, hospitalization, endotracheal intubation, preterm birth, and death [9, 11]. Vaccination definitely prevents affliction by severe COVID-19 and reduces the rate of its severe maternal and fetal complications [46].

Cues to action, i.e., healthcare providers’ recommendations, were another significant factor affecting HACV in the present study. Data from previous pandemics such as the H1N1 pandemic show higher vaccination rate among women who received their physicians’ recommendations about vaccination [47]. Healthcare providers have significant role in providing vaccination-related recommendations to individuals who are at high-risk for infections. Therefore, they need to kindly recommend individuals with HACV to receive the vaccine [48]. Strategies such as motivational interviewing [49] and social media [50] can be used to motivate individuals for vaccination.

Our findings also indicated that perceived barriers had significant relationship with HACV. These barriers are mainly due to women’s concern over the negative effects of vaccination on pregnancy and fetus. Studies showed that woman’s concerns over the effects of vaccination on pregnancy and fetus [51] together with inconsistencies in the recommendations of healthcare organizations respecting the safety of vaccination in pregnancy [45] significantly reduced pregnant women’s trust in COVID-19 vaccine. Media magnification of the negative effects of the COVID-19 vaccination during pregnancy also fueled false beliefs about the vaccine [52, 53]. The side effects of COVID-19 vaccine among pregnant women include pain, headache, fever, myalgia, shivering, and nausea and no serious complication. Women who are concerned with the negative effects of COVID-19 vaccine on their fetus should be ensured that inactivated vaccines such as the influenza vaccine have frequently been used in pregnancy with no serious fetal side effects [54]. In a study, those who may face barriers to health care services were more likely to report vaccine hesitancy [55].

An interesting finding of this study was the insignificant effects of perceived threat on HACV. This finding denotes that pregnant women probably have poor understanding about the risks of affliction by COVID-19 and the greater severity of the disease during pregnancy. This is due to poor educational programs about the effects of COVID-19 during pregnancy. Therefore, quality educational programs are needed to improve pregnant women’s knowledge and awareness of the complications of COVID-19 among pregnant women and the importance of vaccination.

Findings showed that the rate of HACV was high. This is in agreement with the findings of previous studies [56–60]. HACV rate in this study is higher than the rate in the general Iranian population which was 33.5% in a study [61] and 14.7% in another study [62]. This higher HACV among pregnant women is attributable to their concerns over the safety of COVID-19 vaccine for pregnancy and fetus [51, 59–62]. A study reported uncertainty over the safety of COVID-19 vaccine as a major reason for pregnant women’s non-participation in COVID-19 vaccine trials and the paucity of information about the safety of the vaccine for pregnant women [63]. A qualitative study showed that pregnant women considered COVID-19 vaccine more dangerous than affliction by COVID-19 [64]. Such imagination can negatively affect their acceptance of the vaccine.

We also found a significant relationship between COVID-19 knowledge and HACV. Previous studies also reported that the acceptance of COVID-19 vaccine had significant relationship with COVID-19 knowledge [27] and educational level [65]. Individuals with higher educational level usually have more awareness about the different aspects of vaccination. Meanwhile, two studies showed that pregnant women with higher educational level had lower desire to receive vaccine [66, 67]. This is probably due to the fact that those with higher education level have better access to information about the adverse effects of vaccination or information about the unknown effects of vaccination on pregnant women and hence, are less willing to receive COVID-19 vaccine.

We also found insignificantly higher HACV rate among participants who had not received influenza vaccine. A study in the United Kingdom also revealed that HACV rate was two times more among pregnant women who had not received influenza vaccine and four times more among pregnant women who had not received tetanus vaccine [68].

To the best of our knowledge, this was the first study in its kind into HACV among pregnant women in Iran. This study conducted at a time when different vaccines were approved by health organizations, and appropriate information was available about the effects and side effects of vaccines, which influences the views and attitudes towards the COVI-19 vaccine. This study had some limitations. For example, it was a cross-sectional study which assessed the existence of relationships among variables but provided no information about the direction of the relationships. Moreover, data were collected through the self-report method and hence, may be affected by some kinds of bias for example recall bias. Furthermore, participants were from different gestational age groups, while HACV may vary with gestational age. Therefore, longitudinal studies are recommended to assess HACV among pregnant women based on HBM.

## Conclusion

This study suggests high HACV among pregnant women. The perceived benefits and cues to action components of HBM have significant effects on pregnant women’s HACV, while the perceived threat component has no significant effect on their HACV. Educational interventions are needed to improve pregnant women’s awareness of the risks of COVID-19 for pregnant women.

## Data Availability

The datasets analyzed during the current study are available from the corresponding author on reasonable request.

## Abbreviations

COVID-19: Coronavirus disease 2019
HACV: Hesitancy about COVID-19 vaccination
HBM: Health Belief Model
SPSS: Statistical Package for Social Sciences
SD: Standard Deviation
aOR: Adjusted Odd Ratio
CI: Confidence Interval
